# Learning Across Generations: The Dual Benefits of Intergenerational Mentoring for Students and Society

**DOI:** 10.1093/geroni/igaf122.2207

**Published:** 2025-12-31

**Authors:** Emily Ihara, Cathy Tompkins, Madeline McIntyre, Shannon Arnette

**Affiliations:** George Mason University, Fairfax, Virginia, United States; George Mason University, Fairfax, Virginia, United States; Virginia Commonwealth University, Richmond, Virginia, United States; Virginia Commonwealth University, Richmond, Virginia, United States

## Abstract

Despite efforts to encourage careers in aging, fewer than 1% of first-year medical fellows choose geriatrics, and only about 3% of master’s-level social work students enroll in an aging concentration. Intergenerational mentoring programs present a unique opportunity to foster meaningful student-older adult connections, enhance learning outcomes, and challenge societal ageism. Such programs are particularly valuable for medical and social work students, helping them develop empathy, cultural humility, and a deeper understanding of aging to provide effective, person-centered care. This study examines attitudes toward aging among medical (n = 192) and social work (n = 7) students before program implementation. Using a modified COCOA measurement tool (Hollar et al., 2011), the Geriatrics Attitudes Scale (Reuben et al., 1998), the Relational Aging Anxiety Scale (Gendron et al., 2020), and open-ended responses, students reflected on their perceptions of aging and working with older adults post-degree. Preliminary analysis of social work students’ open-ended questions reveals a range of perspectives, from optimism and aging as a source of wisdom to anxiety about physical decline and societal ageism. Among medical students, over 87% agreed they could learn from older adults, but only 14% expressed strong interest in geriatrics. While nearly 60% felt confident working with older adults, only 23% found it deeply fulfilling. Structured mentoring may shift these perspectives by offering experiential learning that challenges biases and enhances professional interest. Future analysis will assess post-program attitude shifts and their impact on aging-related career pathways.

